# Association between Six *CETP* Polymorphisms and Metabolic Syndrome in Uyghur Adults from Xinjiang, China

**DOI:** 10.3390/ijerph14060653

**Published:** 2017-06-18

**Authors:** Huixian Hou, Rulin Ma, Heng Guo, Jia He, Yunhua Hu, Lati Mu, Yizhong Yan, Jiaolong Ma, Shugang Li, Jingyu Zhang, Yusong Ding, Mei Zhang, Qiang Niu, Jiaming Liu, Shuxia Guo

**Affiliations:** Department of Public Health and Key Laboratory of Xinjiang Endemic and Ethnic Diseases of the Ministry of Education, Shihezi University School of Medicine, Shihezi 832002, China; sjzhx06@sina.com (H.H.); marulin@126.com (R.M.); guoheng@shzu.edu.cn (H.G.); hejia123.shihezi@163.com (J.H.); hyh6133@sina.com (Y.H.); murat08123@163.com (L.M.); erniu19880215@sina.com (Y.Y.); jiaojiaolong881202@163.com (J.M.); lishugang@ymail.com (S.L.); yfyxxzjy@126.com (J.Z.); 13399931625@163.com (Y.D.); zmberry@foxmail.com (M.Z.); niuqiang1214@163.com (Q.N.); liujiaming@shzu.edu.cn (J.L.)

**Keywords:** *CETP* gene, polymorphism, metabolic syndrome, Uyghur

## Abstract

Objective: To explore the association between *CETP* gene polymorphisms and metabolic syndrome (MS), as well as the relationship between the *CETP* gene polymorphisms and each component of MS. Methods: A total of 571 individuals which were randomly selected from 5692 Uyghur adults were subdivided into two groups, including 280 patients with MS and 291 control subjects, using the group-matching method after matching for gender. We detected *CETP* polymorphisms (rs5882, rs1800775, rs3764261, rs12149545, rs711752, and rs708272) by using the Snapshot method. Results: (1) Significant differences were found involving the frequency distribution of genotypes and alleles of rs1800775, rs3764261, rs12149545, rs711752, and rs708272 between the control and MS groups (all *p* < 0.05). (2) rs1800775, rs3764261, rs12149545, rs711752, and rs708272 polymorphisms were significantly related to the risk of MS (all *p* < 0.05). (3) The rs1800775 polymorphism was associated with high fasting blood glucose levels and low high density lipoprotein cholesterol (HDL-C); rs3764261 and rs12149545 polymorphisms were associated with all components of MS except high blood pressure; rs711752 and rs708272 polymorphisms were associated with low HDL-C (all *p* < 0.05). (4) Complete linkage disequilibrium (LD) was identified for two pairs of single nucleotide polymorphisms (SNPs) (rs3764261 and rs12149545 (D’ = 1.000, r^2^ = 0.931), rs711752 and rs708272 (D’ = 1.000, r^2^ = 0.996)). (5) The A-G-G-G-C (*p* = 0.013, odds ratio [OR] = 0.622, 95% confidence interval [95% CI] = 0.427–0.906) and A-T-A-A-T (*p* < 0.001, OR = 0.519, 95% CI = 0.386–0.697) haplotypes were more frequent in the control group than in the case group. ***Conclusions***: The rs1800775, rs3764261, rs12149545, rs711752, and rs708272 polymorphisms of *CETP* were associated with MS and its components among the Uyghur ethnic group. Complete LD was found between two pairs of SNPs (rs3764261 and rs12149545, rs711752, and rs708272). The A-G-G-G-C and A-T-A-A-T haplotypes might be protective factors for MS.

## 1. Introduction

Metabolic syndrome (MS), which is perceived as a chronic and complex disease, has become a worldwide public health challenge for its concomitant complications over recent years [1]. Its clinical symptoms include three or more of the following factors: central obesity, raised blood pressure, elevated glucose, high triglyceride (TG) levels, and depressed high density lipoprotein cholesterol (HDL-C) [2]. At present, reaching a consensus on the definition of MS is further complicated by the fact that it is a progressive disorder [3], in that its several components tend to worsen over time. In addition, the prevalence of MS is generally high globally. In the United States, from 2003 to 2012, the overall prevalence of MS was 33% [4]. In Australia, the prevalence of MS was 30.7% [5]. In Japan, the prevalence of MS among 2113 subjects was 22.8% for males and 8.7% for females [6]. In China, a meta-analysis with a total population of 226,653 Chinese subjects from mainland China showed that the pooled prevalence of MS was 24.5% [7]. Our preliminary findings indicated that MS prevalence in the Uyghur population in Xinjiang was 21.2% [8]. This was lower than the 23.3% reported for adults in Northern China through an age-scale epidemiological investigation [9]. Later, another epidemiological survey in Xinjiang also showed a high prevalence of MS among the Uyghur [10]. The age-standardized prevalence of MS was 27.9%, which was slightly lower than that in the Han population in the same area (31.3%). However, the cause of MS remains ambiguous, involving a combination of age, race, genetic factors, diet, behavior, and environmental factors [11]. Genetic factors increasingly attract the attention of scholars’.

Cholesteryl ester transfer protein (*CETP*) is mainly secreted by the spleen, liver, and adipose tissue. *CETP* is considered a key protein for the process of reverse cholesterol transport, and mediates the exchange and transfer of cholesterol esters (CEs) and triglycerides (TGs). CE generated by lecithin cholesterol acyltransferase (LCAT) in HDL is transferred to other lipoproteins by *CETP*. *CETP* promotes the removal of CE from HDL in exchange for TGs derived primarily from very low density lipoprotein (VLDL) or chylomicrons [12]. High *CETP* activity lowers the concentration of HDL-C [13], regulated by the *CETP* gene and the availability of substrates for transfer. The *CETP* gene is highly polymorphic. Some variants in the *CETP* gene are associated with decreased plasma *CETP* protein activity and protein levels, thereby resulting in greater HDL-C concentrations [14,15]. Among the *CETP* polymorphisms, rs708272 has been widely studied. Meta-analyses have shown that carriers of the B_2_ allele, associated with lower *CETP*, have higher HDL-C concentrations than B_1_B_1_ homozygotes [16]. The rs5882 and rs1800775 variants are also related to *CETP* abundance, activity, and lipid levels [17]. At present, the study of the *CETP* gene is mainly related to dyslipidemia. At the same time, the relevance between *CETP* gene polymorphisms and MS components, such as hypertension, type 2 diabetes, and low HDL-C has been studied [18,19,20,21]. We previously demonstrated that five of the eight functional *CETP* polymorphisms (rs1800775, rs3764261, rs12149545, rs711752, and rs708272) are closely linked to dyslipidemia among the Uyghur and Kazakh populations [22]. The studies above showed that *CETP* gene polymorphisms may be related to components of MS. The association between *CETP* and MS has been assessed by many scholars; the results were different in different populations and ethnic groups [23,24], and most were single locus polymorphism studies. Furthermore, there are insufficient data to draw conclusions regarding any association between polymorphisms of *CETP* and MS among the Uyghur ethnicity.

Xinjiang is a multi-ethnic settlement in China, and the Uyghur is the largest population present. It has a unique culture and unique life customs. Because the unique national characteristics and relatively low prevalence of MS, on the basis of previous research, we chose six single nucleotide polymorphisms (SNPs) (rs5882, rs1800775, rs3764261, rs12149545, rs711752, and rs708272) to study associations between *CETP* polymorphisms and MS and its components among the Uyghur. The findings may provide a scientific basis for the study of genetic susceptibility of MS among the Uyghur in Xinjiang.

## 2. Materials and Methods

### 2.1. Study Population

We designed a case-control study that included 571 individuals, resident in Jiashi County, Xinjiang Uyghur Autonomous Region, China, and subdivided into two groups including 280 MS patients and 291 healthy control individuals. All subjects in this study were randomly selected from our previous stratified randomized cluster samples using the group-matching method [25].

### 2.2. Epidemiological Survey and Biochemical Detection

Using a self-developed questionnaire, detailed information on demographic and personal lifestyles for each participant was collected during a face-to-face interview by trained investigators. The questionnaire included personal profile, details of disease, family medical history, diet, current alcohol consumption, smoking status, and physical exercise. Height, weight, waist circumference, hip circumference, and blood pressure were measured by trained field workers according to standardized methods [26]. Biochemical tests of blood samples included tests for fasting TG, total cholesterol (TC), HDL-C, LDL-C, and fasting plasma glucose (FPG). All blood detections were analyzed using an automatic biochemical analyzer (AU400, Olympus: Tokyo, Japan). Each subject signed an approved informed consent form. All experiments were performed in accordance with relevant guidelines and regulations. This study was approved by the Institutional Ethics Review Board (IERB) of the First Affiliated Hospital of Shihezi University School of Medicine (IERB No. SHZ2010LL01).

### 2.3. MS Diagnostic Criteria

MS was diagnosed using a new harmonizing definition formulated by the Joint Interim Statement criteria (JIS criteria) [2], which requires that the participants have three or more of the following five components: (1) central obesity (defined as waist circumference ≥85 cm in male subjects and ≥80 cm in female subjects, Chinese population waist circumference cutoffs); (2) elevated triglycerides: TG >150 mg/dL (1.7 mmol/L); (3) reduced HDL-C: HDL-C <40 mg/dL (1.0 mmol/L) in male subjects and <50 mg/dL (1.30 mmol/L) in female subjects; (4) elevated blood pressure: systolic blood pressure ≥130 mmHg and/or diastolic blood pressure ≥85 mmHg; (5) elevated fasting glucose: fasting plasma glucose ≥100 mg/dL (5.6 mmol/L).

### 2.4. DNA Extraction and Genotyping Analysis

Fasting venous blood (200 µL) was taken from each study subject, and a blood genomic DNA isolation kit (Non-centrifugal columnar, Tiangen, Beijing, China) was used to isolate the genomic DNA. The extracted DNA was verified by gel electrophoresis (0.7% agarose). A NanoDrop spectrophotometer (Thermo Scientific, Waltham, MA, USA) was used for quantitative determination of DNA concentration and purity: concentration ≥30 ng/µL and purity levels (optical density [OD]: OD_260_/OD_280_) of 1.7–2.0 were considered acceptable. Samples that met these criteria were diluted to 10–30 ng/µL using double-distilled water and then stored at −80 °C. The sequences of the forward and reverse primers designed using the Mysequenom tool (www.mysequenom.com/Home) and AssayDesigner3.0 software (Sequenom, Inc., San Diego, CA, USA) are shown in Table 1. The process of polymerase chain reaction (PCR) amplification, purification, and single-base extension were consistent with previous research [22]. All representative SNP genotyping experiments were performed using TaqMan technology on an ABI3730xl system (Applied Biosystems, Foster City, CA, USA). ABI GeneMapper was used to complete the classification and present the results.

### 2.5. Statistical Analysis

We used EpiData3.02 software to establish a database and adopted a double entry method for data input and logic error detection. SPSS statistical software version 17.0 for Windows (SPSS, Inc. Chicago, IL, USA) was used for basic statistical analysis. Non-normally distributed continuous variables such as age, height, weight, body mass index (BMI), systolic blood pressure (SBP), diastolic blood pressure (DBP), TG, TC, HDL-C, and LDL-C were presented as median and interquartile ranges (25th, 75th percentile). We conducted the Wilcoxon rank sum test to compare the differences of the means from two groups of measurement data. Categorical data such as gender, smoking status, and alcohol consumption were evaluated using the chi-square test. To estimate the association between the SNPs in *CETP* and MS, we conducted a logistic regression analysis to evaluate the odds ratios (OR) and 95% confidence intervals (95% CI). MS (0 = no, 1 = yes), central obesity (0 = no, 1 = yes), high blood pressure (0 = no, 1 = yes), high fasting glucose (0 = no, 1 = yes), high TG (0 = no, 1 = yes), and low HDL-C (0 = no, 1 = yes) were used as dependent variables, respectively. Independent variables included age, gender (1 = male, 2 = female), smoking (0 = no, 1 = yes), alcohol consumption (0 = no, 1 = yes), rs5882 (AA = 1, AG = 2, GG = 3), rs1800775 (AA = 1, AC = 2, CC = 3), rs3764261 (GG = 1, GT = 2, TT = 3), rs12149545 (GG = 1, GA = 2, AA = 3), rs711752 (GG = 1, GA = 2, AA = 3), and rs708272 (CC = 1, CT = 2, TT = 3). SHEsis software was used to perform the Hardy–Weinberg equilibrium test, haplotype construction, and statistical analysis [27]. The criterion for significance was set at *p* < 0.05 for all tests.

## 3. Results

### 3.1. Assessment of Demographic and Clinical Characteristics of Study Subjects

Table 2 presents the demographic and clinical characteristics of the study population. The distribution of gender and height were well-matched (*p* > 0.05). The average age, weight, BMI, waist circumference, hip circumference, blood pressure, FPG, TG, TC, and LDL-C levels were higher in the case group than the control group (all *p* < 0.001), while the HDL-C levels were lower in the case group. There was no significant difference on current smokers and current alcohol drinkers between the two groups (*p* > 0.05).

### 3.2. Genotype and Allele Frequencies Distribution

The six SNPs genotypes and alleles frequency distributions for *CETP* between the control and case group are shown in Table 3. No obvious difference was found regarding the frequency distribution of the genotypes of rs5882 between the two groups (*p* > 0.05). We were pleasantly surprised to find that there were significant differences for the other five SNPs between the two groups (all *p* < 0.05). Similar to the allele frequency distribution analysis result, no obvious difference was found for rs5882 between the two groups (*p* > 0.05). After further investigation, we found that the T allele of rs3764261 (24.6 vs. 36.3, *p* < 0.001, OR = 0.575, 95% CI = 0.445–0.743) and rs708272 (42.1 vs. 51.2, *p* = 0.002, OR = 0.694, 95% CI = 0.550–0.877), and the A allele of rs12149545 (22.5 vs. 35.4, *p* < 0.001, OR = 0.530, 95% CI = 0.408–0.688) and rs711752 (42.3 vs. 51.2, *p* = 0.003, OR = 0.699, 95% CI = 0.554–0.883) had a lower frequency in the case group than in the controls. However, we observed that rs1800775-C more frequently appeared in the case group than in the control group (45.7 vs. 38.3, *p* = 0.011, OR = 1.356; 95% CI = 1.071–1.716). We also calculated the Hardy–Weinberg equilibrium *p*-value for each SNP to evaluate the sample’s representativeness. Six *CETP* SNPs were in the Hardy–Weinberg equilibrium (all *p* > 0.05).

### 3.3. Association of Six SNPs and MS Subjects

We conducted multivariate logistic regression analysis to detect any associations between six *CETP* SNPs and MS. The results are shown in Table 4. The logistic regression analysis confirmed the results of the χ^2^ test of independence on the genotype frequencies of the *CETP* polymorphisms between cases and controls, following adjustment for age, gender, smoking, and alcohol consumption.

### 3.4. Risk Factor Analysis between Six CETP SNPs and Five Components of MS

We detailed a risk factor analysis between the six *CETP* SNPs and five components in MS after finding the association of six SNPs and MS subjects (Table 5). SNP rs5882 had no effect on the five components of MS (all *p* > 0.05). For rs1800775, compared with AA genotype carriers, rs1800775-AC (*p* = 0.026, OR = 0.540, 95% CI = 0.314–0.929) and rs1800775-CC (*p* = 0.043, OR = 0.448, 95% CI = 0.206–0.976) carriers had a lower risk of high fasting glucose, whereas rs1800775-AC (*p* = 0.031, OR = 1.557, 95% CI = 1.041–2.330) and rs1800775-CC (*p* = 0.003, OR = 2.205, 95% CI = 1.316–3.696) carriers were at a higher risk of low HDL-C. For rs3764261, the risk of rs3764261-GT carriers in central obesity (*p* = 0.004, OR = 0.631, 95% CI = 0.460–0.865) and high TG (*p* = 0.022, OR = 0.667, 95% CI = 0.472–0.943) patients were lower than that of rs3764261-GG carriers, while rs3764261-GT (*p* = 0.001, OR = 2.652, 95% CI = 1.526–4.609) carriers had a higher risk of high fasting glucose. For rs12149545, the risk of rs12149545-AG carriers in central obesity (*p* = 0.001, OR = 0.597, 95% CI = 0.435–0.819), high TG (*p* < 0.001, OR = 0.587, 95% CI = 0.413–0.833) and low HDL-C (*p* = 0.018, OR = 0.633, 95% CI = 0.434–0.924) patients was lower than that of rs12149545-GG carriers, while rs12149545-AG (*p* = 0.006, OR = 2.125, 95% CI = 1.245–3.629) carriers had a higher risk of high fasting glucose. For rs711752, compared with the GG genotype, rs711752-AA (*p* = 0.007, OR = 0.500, 95% CI = 0.303–0.824) carriers had a lower risk of low HDL-C. Compared with rs708272-CC, rs708272-TT (*p* = 0.006, OR = 0.498, 95% CI = 0.302–0.821) might reduce the risk of low HDL-C.

### 3.5. Linkage Disequilibrium (LD) and Hardy–Weinberg Equilibrium Testing

In our study, we calculated pairwise linkage disequilibrium (LD) between six SNPs, and the result is shown in Table 6. The value of D’ ranged from 0.060 to 1.000, and the value of r^2^ spanned 0.003 to 0.996. Complete LD was observed for two pairs of SNPs: rs3764261 and rs12149545 (D’ = 1.000, r^2^ = 0.931) and rs711752 and rs708272 (D’ = 1.000, r^2^ = 0.996).

### 3.6. Haplotype Analysis

Since rs5882 was obviously not in LD with the other SNPs, it was excluded during the process of haplotype construction. The remaining five SNPs above formed 11 kinds of haplotypes among the 32 types of possible haplotypes identified through SHEsis software. The results of four haplotypes analyses, in which frequencies were greater than 0.001, are presented in Table 7. The global haplotype frequencies in the case group were significantly different from the control group (*p* < 0.001). The A-G-G-G-C (*p* = 0.013, OR = 0.622, 95% CI = 0.427–0.906) and A-T-A-A-T (*p* < 0.001, OR = 0.519, 95% CI = 0.386–0.697) haplotypes were more frequent in the controls than in the case group. Compared with the C-G-G-G-C haplotype, A-G-G-G-C and A-T-A-A-T haplotypes might reduce the risk of MS.

## 4. Discussion

In China, the prevalence of MS is generally high. The rate in the Uyghur ethnic group was lower than that of Han and Kazak populations in the same area [10,28]. This may be related to Uyghur characteristics and genetic susceptibility. Therefore, we chose the Uyghur to study any relationships between *CETP* gene polymorphisms and MS. Since *CETP* is a key factor in the process of reverse cholesterol transport (RCT) and plays an important role in lipoprotein metabolism, it has attracted the attention of many scholars [29]. Some studies proved that *CETP* mutations may affect the abundance of serum *CETP*, which, in turn, affects lipid metabolism [17].

The results in this study showed that the levels of average age, BMI, waist circumference, blood pressure, FPG, and TG were higher in the case group than in the controls, while the level of HDL-C was lower in cases than in the control group. Some studies showed that the risk of MS increases with age [30], so the variable of age should be adjusted for in risk factor analysis. Central obesity, elevated TG, reduced HDL-C, elevated blood pressure, and elevated fasting glucose are clinical symptoms of MS. Consistent with other studies [31], the levels of TC and LDL-C in the case group were higher than in the controls. Other studies [32] have shown that normal or slightly elevated LDL-C levels are characteristics of dyslipidemia among MS patients. However, differing results have appeared in other research [33]. This might be caused by ethnic differences. The distribution of gender between the two groups was well matched, and SNPs were all in accordance with Hardy–Weinberg equilibrium. The results above indicated that the genotypic frequencies were representative of their respective populations.

No obvious difference was found between the control and MS group including the frequency distribution of the genotypes and alleles of rs5882. Combined with the results of regression analysis, this SNP may not be involved in MS among the Uyghur population for the ethnic difference. Furthermore, meta-analyses [17] showed that rs5882 was related to the *CETP* abundance, activity, or lipid levels. Hence, further study is needed. For the other five SNPs, significant differences were found regarding the frequency distribution of genotypes and alleles between the control and MS groups. Our research showed that rs1800775-C frequency (41.9%) was lower than that in the Latvian (44.3%) [21] and European (51%) [34]. Compared with rs1800775-A carriers, individuals who carried rs1800775-C were more likely to develop MS. This SNP may increase the risk of MS. This is consistent with Radovica et al. [21], who also hold the view that rs1800775 can increase the risk of low HDL-C levels. For rs3764261, we discovered statistically significant association between T allele and MS. Furthermore, the rs3764261-T frequency (30.6%) was higher in Uyghur individuals than that in the Han population (16%) [35]. For the rs12149545 and rs711752 polymorphisms, the risk of MS was reduced among the A allele carriers. The rs12149545-A frequency was 29.1%, and the rs711752-A frequency (46.8%) was similar to that in Latvians (46.2%) [21]. For rs708272, the rs708272-T frequency (46.8%) was higher than that in Austria (41.3) [36], Turkey (43%) [37], or Southern Thailand (37.43%) [38]. Moreover, rs708272-T plays an inactive role in the development of MS. This is consistent with Anton et al. [36] who hold that the risk of MS was reduced by 32% (*p* = 0.005, OR = 0.68) in carriers of the B_2_ variant. However, this was different from observations by Jeenduang [38] and Maroufi [39].

Based on these findings, to verify the authenticity, we conducted a logistic regression analysis adjusting for covariates such as age, gender, smoking, and alcohol consumption. We found that the association remained. The results showed that rs3764261-GT/TT, rs12149545-AG/AA, rs711752-AA, and rs708272-TT might reduce the risk of MS on different levels. This suggested that the four SNPs may be associated with MS in the Uyghur population. The rs708272 polymorphism was one of the loci with high variant frequency, and it is the most widely studied *CETP* gene polymorphism. Compared with the CC genotype, the risk of MS among the rs708272-TT carriers was reduced. Similarly, a previous study showed that MS patients have a higher prevalence of the B_1_B_1_(CC) genotype in Egypt [40]. This also explained why the prevalence of MS among the Uyghur is lower than that among other ethnic groups in the same area [10]. However, compared with AA genotype carriers, rs1800775-CC increased the risk of MS. Hence, we suspected that rs1800775-CC might be a risk factor for MS. The association of rs1800775 with MS has not been previously reported, while the relationship between rs1800775 and low HDL-C had been well established in a systematic in-depth review [41]. It has the potential to increase the risk of low HDL-C and may serve as a basis for MS. These studies above indicated that the correlation between *CETP* gene polymorphisms and MS has racial and ethnic differences.

After analyzing the association between six SNPs and MS, we further investigated the relationship between the six SNPs and five components of MS, respectively. Compared with the relevance between six SNPs and MS, we found that the relevance was slightly different among the five components. For central obesity, we found that rs3764261-GT and rs12149545-AG might reduce the risk of this disease. However, in another adult Chinese population, Ruan et al. [42] discovered that less common alleles of Taq1b (rs708272) and I405V (rs5882) polymorphisms of *CETP* are moderately associated with risk of obesity. For high blood pressure, no significant correlation was found among the six SNPs. For high fasting glucose, rs1800775-AC/CC might reduce the risk of this disease, but rs3764261-GT and rs12149545-AG might increase the risk. However, another study showed that the rs3764261 polymorphism of *CETP* is not associated with type 2 diabetes in patients with clinically manifest vascular disease [43]. For high TG, rs3764261-GT/TT and rs12149545-AG might reduce the risk of elevated TG. However, a statistically significant association was not discovered between rs3764261 and high TG in another Chinese population [44]. For low HDL-C, we found that all of the SNPs we studied, except rs5882, had an effect on the risk of low HDL-C. The rs1800775-AC/CC increased the risk of low HDL-C, while the other four SNPs reduced the risk of low HDL-C. In accordance with our observation, the effect of the six SNPs on the risk of low HDL-C was previously verified in other populations [21,22,37,42,43,45,46]. Combined with functional *CETP* gene variants effects and serum HDL-C concentration [34,47], we suspected that low HDL-C might be the most relevant component to *CETP* gene polymorphisms among the five components of MS.

Complete LD was found for two pairs of SNPs, and strong LD was identified between other SNPs. Similarly, strong LD was found between rs708272 and rs1800775 in Europeans [48] and in other parts of China [49]. Some reports suggest that the rs708272 polymorphism acts through LD to a second SNP in the promoter of the *CETP* gene at position rs1800775 from the transcription start site [50]. Therefore, the discovery of complete LD may provide direction for further studies.

The association of four haplotypes of the *CETP* gene and MS disease was analyzed using SHEsis software. We observed that the A-G-G-G-C and A-T-A-A-T haplotypes were more frequent in the controls than in the case group. Thus, we suspected that the two haplotypes, especially the A-T-A-A-T haplotype, were protective factors that can reduce the risk of MS and protect people from the harm of MS. Similarly, the common A-A-T haplotype defined by the G-2708A, rs1800775, and rs708272 polymorphisms was consistently associated with reduced *CETP* activity and increased HDL-C levels in another study [51]. However, haplotype formation was influenced by many aspects and we just demonstrated *CETP* gene SNP haplotypes in a small random sample of the Uyghur population. Therefore, this complex relationship needs to be further explored and verified.

## 5. Study Limitations

Despite our comprehensive analysis, there are still potential limitations in our study. First, more than 180 SNPs exist in the *CETP* gene. In our study, we only selected six major functional SNPs to study the relationship between *CETP* and MS. Therefore, the analysis and results of our study may be unilateral. In addition, we investigated the association of six SNPs with MS and its components, but did not study the interaction between genes and genes, or between genes and environmental factors in MS. Therefore, we need further research into the effects of these interactions.

## 6. Conclusions

The rs1800775, rs3764261, rs12149545, rs711752, and rs708272 polymorphisms of *CETP* were associated with MS and its components among the Uyghur ethnic group. Complete LD was found for two pairs of SNPs (rs3764261 and rs12149545, and rs711752 and rs708272). The A-G-G-G-C and A-T-A-A-T haplotypes might be protective factors for MS.

## Figures and Tables

**Table 1:** Sequences of forward and reverse primers for genotyping of the *CETP* gene.

Single Nucleotide Polymorphisms (SNPs)	Forward Sequence	Reverse Sequence	PCR Product	Allele
rs5882	TCACCATGGGCATTTGATTGG	TATCAATGACTGGGAAGAGGG	211 bp	A/G
rs1800775	CCAGCTGTAGGTAAAGTACTG	CAGTCCTCTATGTAGACTTTCC	210 bp	A/C
rs3764261	AGCCACCATGCCTGGCCTATG	GCTTCCATGACCCCAAGCCTC	360 bp	G/T
rs12149545	AGCCACCATGCCTGGCCTATG	GCTTCCATGACCCCAAGCCTC	360 bp	G/A
rs711752	GCCTCCGTCACCTGAGCTCATG	GATGGGCTGAGTGGAGCTGTCA	276 bp	G/A
rs708272	GCCTCCGTCACCTGAGCTCATG	GATGGGCTGAGTGGAGCTGTCA	276 bp	C/T

**Table 2:** Demographic and clinical characteristics of study subjects.

Characteristics	Control (*n* = 291)	Case (*n* = 280)	Z/χ^2^	*p*
Male/female	152/139	140/140	0.285	0.593
Age, years	40 (29–56)	50 (38–60)	−4.914	*p* < 0.001
Height, cm	158 (153–166)	160 (153–167)	−1.066	0.286
Weight, kg	55 (48–60.5)	64 (55–73)	−10.002	*p* < 0.001
BMI, kg/m^2^	21.03 (19.63–23.03)	24.79 (22.20–27.64)	−12.065	*p* < 0.001
Waist circumference, cm	78 (74–82)	91.5 (85–99)	−15.706	*p* < 0.001
Hip circumference, cm	92 (88–96)	99 (94–104)	−11.341	*p* < 0.001
SBP, mmHg	118 (110–126)	132 (120–150)	−10.118	*p* < 0.001
DBP, mmHg	74 (68–80)	82 (78–92)	−8.346	*p* < 0.001
FPG, mmol/L	4.24 (3.85–4.69)	4.63 (4.11–5.25)	−6.310	*p* < 0.001
TG, mmol/L	0.78 (0.58–0.96)	2.31 (1.78–3.01)	−17.678	*p* < 0.001
TC, mmol/L	4.15 (3.65–4.60)	4.75 (4.00–5.63)	−6.980	*p* < 0.001
HDL-C, mmol/L	1.34 (1.23–1.49)	0.90 (0.78–1.02)	−17.940	*p* < 0.001
LDL-C, mmol/L	2.15 (1.80–2.62)	2.66 (2.05–3.22)	−6.258	*p* < 0.001
Current smoker, *n* (%)	40 (13.7)	36 (12.9)	0.098	0.755
Current alcohol drinker, *n* (%)	7 (2.4)	9 (3.2)	0.343	0.558

**Notes:** BMI: body mass index; SBP: systolic blood pressure; DBP: diastolic blood pressure; FPG: fasting plasma glucose; TG: triglyceride; TC: total cholesterol; HDL-C: high-density lipoprotein-cholesterol; LDL-C, low-density lipoprotein-cholesterol.

**Table 3:** Genotype and allele frequencies for the six *CETP* SNPs between control and case group and Hardy–Weinberg equilibrium testing.

SNPs	Group	Genotype Distribution *n* (%)					HWE-P	Allelic Distribution			
		CC	CR	RR	χ^2^	*p*		MAF *n* (%)	χ^2^	*p*	OR (95% CI)
rs5882	Control	122 (41.9)	127 (43.6)	42 (14.4)	0.489	0.783	0.341	R: 211 (36.3)	0.400	0.527	1.000
	Case	122 (43.6)	123 (43.9)	35 (12.5)			0.645	R: 193 (34.5)			0.925 (0.725–1.179)
rs1800775	Control	111 (38.1)	137 (47.1)	43 (14.8)	6.388	0.041	0.945	R: 223 (38.3)	6.415	0.011	1.000
	Case	85 (30.4)	134 (47.9)	61 (21.8)			0.549	R: 256 (45.7)			1.356 (1.071–1.716)
rs3764261	Control	114 (39.2)	143 (49.1)	34 (11.7)	18.966	*p* < 0.001	0.323	R: 211 (36.3)	18.132	*p* < 0.001	1.000
	Case	156 (55.7)	110 (39.3)	14 (5.0)			0.840	R: 138 (24.6)			0.575 (0.445–0.743)
rs12149545	Control	117 (40.2)	142 (48.8)	32(11.0)	23.911	*p* < 0.001	0.253	R: 206 (35.4)	23.015	*p* < 0.001	1.000
	Case	167 (59.6)	100 (35.7)	13 (4.6)			0.687	R: 126 (22.5)			0.530 (0.408–0.688)
rs711752	Control	69 (23.7)	146 (50.2)	76 (26.1)	8.998	0.011	0.945	C: 284 (48.8)	9.040	0.003	1.000
	Case	93 (33.2)	137 (48.9)	50 (17.9)			0.971	R: 237 (42.3)			0.699 (0.554–0.883)
rs708272	Control	69 (23.7)	146 (50.2)	76 (26.1)	9.346	0.009	0.945	C: 284 (48.8)	9.410	0.002	1.000
	Case	94 (33.6)	136 (48.6)	50 (17.9)			0.947	R: 236 (42.1)			0.694 (0.550–0.877)

**Notes:** C–R: rs5882 (A–G), rs1800775 (A–C), rs3764261 (G–T), rs12149545 (G–A), rs711752 (G–A), rs708272 (C–T); HWE-P: Hardy-Weinberg equilibrium *p*-value; MAF: minor allele frequency.

**Table 4:** The relationship between six *CETP* SNPs and metabolic syndrome (MS).

SNP	Genotype	B	SE	Wals χ^2^	df	*p*	OR	95% CI
rs5882	AA						1	
	AG	−0.099	0.185	0.285	1	0.593	0.906	0.630–1.303
	GG	−0.120	0.268	0.200	1	0.654	0.887	0.524–1.501
rs1800775	AA						1	
	AC	0.238	0.193	1.518	1	0.218	1.269	0.869–1.854
	CC	0.654	0.252	6.752	1	0.009	1.922	1.174–3.147
rs3764261	GG						1	
	GT	−0.594	0.181	10.714	1	0.001	0.552	0.387–0.788
	TT	−1.242	0.350	12.605	1	*p* < 0.001	0.289	0.145–0.573
rs12149545	GG						1	
	AG	−0.725	0.182	15.783	1	*p* < 0.001	0.484	0.339–0.693
	AA	−1.284	0.360	12.743	1	*p* < 0.001	0.277	0.137–0.560
rs711752	GG						1	
	AG	−0.329	0.202	2.634	1	0.105	0.720	0.484–1.071
	AA	−0.702	0.247	8.054	1	0.005	0.496	0.305–0.805
rs708272	CC						1	
	CT	−0.344	0.202	2.899	1	0.089	0.709	0.477–1.053
	TT	−0.712	0.247	8.288	1	0.004	0.491	0.302–0.797

**Notes:** B: regression coefficient; SE: Standard error; OR: odds ratio; CI: confidence interval. The rs5882-AA, rs1800775-AA, rs3764261-GG, rs12149545-GG, rs711752-GG, and rs708272-CC were used as reference genotypes for the risk analysis.

**Table 5:** Risk factor analysis between six *CETP* SNPs and five components in MS.

SNP		Central Obesity		High Blood Pressure		High Fasting Glucose		High TG		Low HDL-C	
		*p*	OR (95% CI)	*p*	OR (95% CI)	*p*	OR (95% CI)	*p*	OR (95% CI)	*p*	OR (95% CI)
rs5882	AA		1		1		1		1		1
	AG	0.549	0.906 (0.657–1.250)	0.376	0.854 (0.601–1.212)	0.933	0.977(0.572–1.670)	0.693	1.073(0.755–1.525)	0.151	0.754(0.513–1.109)
	GG	0.953	0.986 (0.624–1.560)	0.481	1.194 (0.729–1.955)	0.947	0.974(0.441–2.150)	0.121	0.663(0.395–1.115)	0.489	0.827(0.483–1.416)
rs1800775	AA		1		1		1		1		1
	AC	0.864	1.030 (0.738–1.435)	0.498	0.882 (0.615–1.267)	0.026	0.540(0.314–0.929)	0.285	1.224(0.845–1.773)	0.031	1.557(1.041–2.330)
	CC	0.255	1.286 (0.834–1.985)	0.874	1.038 (0.653–1.650)	0.043	0.448(0.206–0.976)	0.093	1.491(0.936–2.377)	0.003	2.205(1.316–3.696)
rs3764261	GG		1		1		1		1		1
	GT	0.004	0.631 (0.460–0.865)	0.972	0.994 (0.707–1.398)	0.001	2.652(1.526–4.609)	0.022	0.667(0.472–0.943)	0.061	0.699(0.481–1.017)
	TT	0.074	0.624 (0.372–1.047)	0.161	0.664 (0.375–1.177)	0.550	1.339(0.514–3.490)	0.042	0.539(0.297–0.978)	0.013	0.443(0.234–0.841)
rs12149545	GG		1		1		1		1		1
	AG	0.001	0.597 (0.435–0.819)	0.953	1.010 (0.717–1.423)	0.006	2.125(1.245–3.629)	*p* < 0.001	0.587(0.413–0.833)	0.018	0.633(0.434–0.924)
	AA	0.070	0.615 (0.363–1.041)	0.059	0.566 (0.313–1.023)	0.680	1.220(0.475–3.135)	0.078	0.583(0.320–1.063)	0.007	0.410(0.214–0.788)
rs711752	GG		1		1		1		1		1
	AG	0.090	0.738 (0.519–1.049)	0.717	1.072 (0.735–1.563)	0.089	1.699(0.923–3.125)	0.146	0.756(0.518–1.103)	0.364	0.824(0.543–1.251)
	AA	0.194	0.758 (0.499–1.151)	0.407	0.826 (0.526–1.297)	0.499	1.291(0.616–2.703)	0.061	0.646(0.409–1.020)	0.007	0.500(0.303–0.824)
rs708272	CC		1		1		1		1		1
	CT	0.076	0.728 (0.512–1.034)	0.608	1.104 (0.757–1.609)	0.082	1.718(0.934–3.162)	0.126	0.745(0.511–1.086)	0.351	0.820(0.540–1.245)
	TT	0.181	0.752 (0.495–1.142)	0.451	0.841 (0.536–1.320)	0.487	1.300(0.620–2.723)	0.056	0.641(0.406–1.011)	0.006	0.498(0.302–0.821)

**Notes:** All the regression analysis was carried out after adjustment for age, gender, smoking, and alcohol drinking. Analysis of high blood pressure and high fasting glucose was then carried out after adjustment for central obesity. The analysis involving high TG was carried out after adjustment for central obesity and low HDL-C. Analysis concerning low HDL-C was carried out after adjustment for central obesity and high TG. The cut-off values (low-high) for the five parameters were as follows: central obesity: waist circumference ≥85 cm in male subjects and ≥80 cm in female subjects; (2) high TG: TG > 150 mg/dL (1.7 mmol/L); (3) low HDL-C: HDL-C < 40 mg/dL (1.0 mmol/L) in male subjects and <50 mg/dL (1.30 mmol/L) in female subjects; (4) elevated blood pressure: SBP ≥130 mm Hg and/or DBP ≥85 mm Hg; (5) high fasting glucose: fasting plasma glucose ≥ 100 mg/dL (5.6 mmol/L). The rs5882-AA, rs1800775-AA, rs3764261-GG, rs12149545-GG, rs711752-GG, and rs708272-CC were used as reference genotypes for the risk analysis of each component.

**Table 6:** Linkage disequilibrium test for the six *CETP* SNPs.

SNP	rs5882	rs1800775	rs3764261	rs12149545	rs711752	rs708272
rs5882	-	0.545	0.069	0.060	0.455	0.457
rs1800775	0.117	-	0.860	0.862	0.833	0.833
rs3764261	0.004	0.235	-	1.000	0.880	0.874
rs12149545	0.003	0.220	0.931	-	0.979	0.972
rs711752	0.129	0.442	0.387	0.446	-	1.000
rs708272	0.130	0.440	0.383	0.441	0.996	-

**Notes:** The upper triangle is D’ value and the lower triangle is r^2^ value.

**Table 7:** The discrepancy of haplotype frequencies of five *CETP* SNPs between the control and case group in the Uyghur.

Haplotype	Case (Freq)	Control (Freq)	χ^2^	*p*	OR (95% CI)
C-G-G-G-C	245.72 (0.439)	193.44 (0.332)			1
A-G-G-A-T	103.92 (0.186)	83.76 (0.144)	0.027	0.868	0.971 (0.689–1.370)
A-G-G-G-C	65.27 (0.117)	81.99 (0.141)	6.176	0.013	0.622 (0.427–0.906)
A-T-A-A-T	123.00 (0.220)	186.19 (0.320)	19.113	*p* < 0.001	0.519 (0.386–0.697)

**Notes:** Global chi2 is 23.149 (frequency <0.03 in both control & case has been dropped.); global *p* < 0.001. The C-G-G-G-C was used as a reference haplotype for obtaining the odds ratio calculations. The order of five SNPs in haplotypes is (from left to right): rs1800775 (A/C), rs3764261 (G/T), rs12149545 (G/A), rs711752 (G/A), and rs708272 (C/T).

## Supplementary Materials

Supplementary File 1
